# A Framework for Understanding the Evasion of Host Immunity by *Candida* Biofilms

**DOI:** 10.3389/fimmu.2018.00538

**Published:** 2018-03-16

**Authors:** Josselyn E. Garcia-Perez, Lotte Mathé, Stephanie Humblet-Baron, Annabel Braem, Katrien Lagrou, Patrick Van Dijck, Adrian Liston

**Affiliations:** ^1^Laboratory of Translational Immunology, VIB Center for Brain and Disease Research, Leuven, Belgium; ^2^Department of Microbiology and Immunology, University of Leuven, Leuven, Belgium; ^3^Center for Microbiology VIB – KU Leuven, Heverlee, Belgium; ^4^Department of Materials Engineering (MTM), KU Leuven, Heverlee, Belgium; ^5^Surface and Interface Engineered Materials, Department of Materials Engineering (MTM), KU Leuven, Heverlee, Belgium; ^6^Clinical Department of Laboratory Medicine and National Reference Center for Mycosis, University Hospitals Leuven, Leuven, Belgium

**Keywords:** biofilms, *Candida*, cytokines, trained immunity, immune resistance

## Abstract

*Candida* biofilms are a major cause of nosocomial morbidity and mortality. The mechanism by which *Candida* biofilms evade the immune system remains unknown. In this perspective, we develop a theoretical framework of the three, not mutually exclusive, models, which could explain biofilm evasion of host immunity. First, biofilms may exhibit properties of immunological silence, preventing immune activation. Second, biofilms may produce immune-deviating factors, converting effective immunity into ineffective immunity. Third, biofilms may resist host immunity, which would otherwise be effective. Using a murine subcutaneous biofilm model, we found that mice infected with biofilms developed sterilizing immunity effective when challenged with yeast form *Candida*. Despite the induction of effective anti-*Candida* immunity, no spontaneous clearance of the biofilm was observed. These results support the immune resistance model of biofilm immune evasion and demonstrate an asymmetric relationship between the host and biofilms, with biofilms eliciting effective immune responses yet being resistant to immunological clearance.

## Introduction

*Candida albicans* can exist in both a unicellular yeast form and a colonial biofilm form. The majority of diseases caused by *C. albicans* include the formation of such a biofilm (1–4). Biofilms shield the fungi from environmental factors and are associated with poor immune clearance by the host (5). *C. albicans* can potentially form biofilm on all implanted medical devices (6, 7), and, as the host immune system appears incapable of eradicating biofilms, removal of the devices is often required (8). In addition, *C. albicans* can also form biofilms in mucosal surfaces such as the vaginal cavity (vulvovaginal candidiasis). These infections are typically not spontaneously cleared by the immune system and usually require antifungals (9–11). Immune evasion of *Candida* biofilms therefore incurs a large public health burden and socioeconomic costs.

### Host Clearance Mechanisms of Yeast Form *Candida* Infections

**Figure 1:**
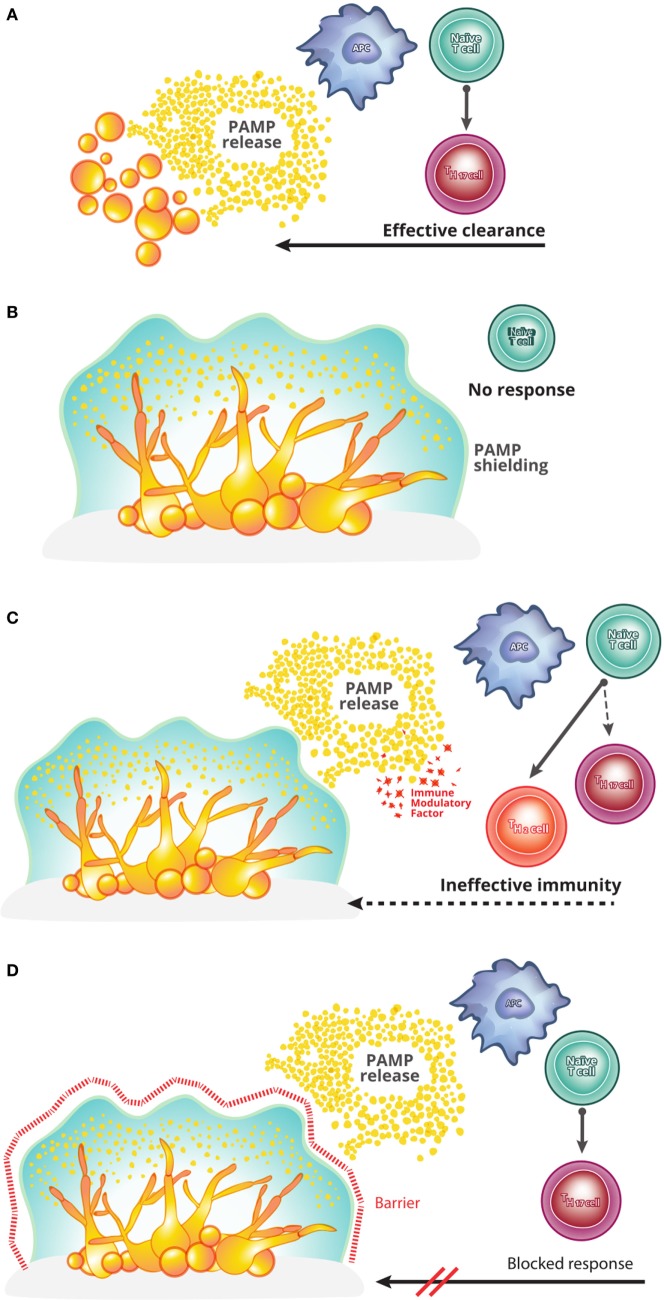
A theoretical framework for understanding *Candida* biofilm immune evasion. **(A)** In a healthy host, infection with the yeast form of *Candida albicans* causes pathogen-associated molecular pattern (PAMPs) release and drives the induction of Th17 cells. Th17 cells, in turn, coordinate an effective host anti-pathogen response, clearing the infection. This process is seemingly inoperative in a *Candida* biofilm growth, for unknown reasons. **(B)** The first model capable of explaining the immune evasion of *C. albicans* biofilms is one of immunological silence. In this model, biofilm structure prevents the release of PAMPs and thereby prevents the initiation of T cell activation and polarization into the effective Th17 lineage. **(C)** The second model to potentially explain biofilm immune evasion is that of immunological deviation. In this model, while the biofilm causes the release of PAMPs and thus the activation of a T cell response against *C. albicans*, additional factors are produced which deviate the responding T cells from an effective Th17 program into an ineffective (e.g., Th2) program. The resulting host immunity is therefore unable to clear the infection. **(D)** The final model capable of explaining the persistence of *C. albicans* biofilms is that of immune resistance. In this model, even in cases where an effective Th17 anti-*Candida* response is initiated, the biofilm remains resistant to the host immunity (e.g., exclusion of effector cells from the biofilm).

Being a commensal, *C. albicans* has a remarkable capacity of adaptation to different niches within its host. However, despite the near ubiquity of commensal *Candida*, invasive infection by yeast form is rare. The immunological response that normally maintains *C. albicans* as a commensal rather than a pathogen is coordinated by Th17 cells (Figure 1A). The Th17 basis of immunity is revealed through the study of congenital forms of *Candida* susceptibility, which, despite representing only a tiny fraction of *Candida* infections, inform as to the immunological pathways essential for preventing infection. Evidence of Th17 involvement in anti-*Candida* host defense comes from a diverse group of patients. Patients with deficiencies in the autoimmune regulator gene (AIRE) present with an autosomal recessive syndrome called autoimmune polyendocrinopathy syndrome type 1 (APS1), of which chronic mucocutaneous candidiasis (CMC) is a key feature. A subset of patients with APS1 has high titers of neutralizing autoantibodies against IL-17A, IL-17F, and IL-22, but not against other cytokines, which correlates with the subset of patients that develop CMC (12, 13). Furthermore, Puel et al. described a loss of function mutation in the IL-17RA, which causes an inability to signal in response to IL-17A and IL-17F, creating a Th17 deficiency and a susceptibility to CMC (14). In addition to the requirement for an effective Th17 response, mannose-binding lectin (MBL) is important for controlling *Candida* infections. MBL deficiencies are associated with susceptibility to *Candida* infections, where deficient patients with lower circulating levels of MBL are susceptible to fungal infections, such as *Candida* vaginitis (15). In the presence of healthy MBL and Th17 levels, fulminant infection with the yeast form of *C. albicans* is rare, indicating the effectiveness of this immunological pathway.

### A Theoretical Framework for Understanding Host Immune Evasion by *Candida* Biofilms

As the majority of infections caused by *C. albicans* are associated with biofilm formation, it is critical to understand the interaction between the host immune system and *C. albicans* biofilms. While the yeast form of *C. albicans* is rapidly cleared from healthy individuals, once a biofilm has developed (e.g., during vulvovaginal candidiasis or on implanted medical devices), it is capable of long-term evasion of host immunity. Here, we propose three basic models that are each capable of explaining the phenomenon of host immunity evasion by *Candida* biofilms. These models are not mutually exclusive and together cover the majority of mechanistic scenarios that are hypothetically capable of explaining the observed evasion.

The first theoretical model for host immune evasion is that of immunological silence (Figure 1B). This model was proposed by Nett and colleagues to explain the paucity of infiltrating leukocytes into the biofilm site (16, 17). This model covers a diverse set of potential scenarios in which the *Candida* biofilm remains “hidden” from the host immune system, with a failure to activate host anti-*Candida* pathways. A definitive example of such a mechanism would be shielding of the biofilm by the biofilm matrix, preventing the release of pathogen-associated molecular patterns and microbial antigens. Such as shielding would ensure that the host immune system remained ignorant of biofilm presence. A potential difficulty of the immunological silence models is the clinical observation of concurrent biofilm and commensal colonizations. As the latter is actively controlled by host immunity, a model relying on immunological silence must include a proviso that the silence only needs to be local in scope, rather than global, with the activation of effective immunity by commensals unable to extend to the site of biofilm infection.

The second theoretical model capable of explaining biofilm evasion of host immunity is that of immune deviation (Figure 1C). Netea and colleagues have proposed a model whereby the biofilm is capable of producing factors that deviate the immune response into an ineffective format (18, 19). In principle, such factors could directly (i.e., a direct impact on leukocytes) or indirectly (e.g., induce the production of host immunomodulatory factors) drive immune deviation. This model is supported by the observation that *C. albicans* cell wall components can induce IL-10 expression (18, 19), known to be effective at supporting Th2 responses at the expense of Th17 responses (20). In this scenario, anti-*Candida* T cells would be activated; however, the deviation into a Th2 response would result in an ineffective immune response, allowing the biofilm to evade clearance.

The third theoretical model to explain long-term survival of the biofilm in the host is that of immune resistance (Figure 1D). This model needs to invoke neither immune silence nor immune deviation, i.e., it is capable of explaining biofilm immune evasion even in the presence of a biofilm-induced effective anti-*Candida* response. Instead, this model postulates that the structural–mechanical or molecular features of the biofilm may render the biofilm-associated *Candida* resistant to immune clearance. Mechanisms such as immune exclusion from the biofilm or a cellular resistance to toxic immune mediators would be covered under this model. This immune resistance model is analogous to the role of the biofilm matrix in *Candida* resistance to antifungal drugs (19, 21, 22).

Despite the sharp theoretical division between these models, existing data are still compatible with each model. For example, the paucity of infiltrating leukocytes in the *Candida* biofilm (16) could be due to either immune silence, preventing immune triggering and thus relying on passive exclusion, immune deviation, producing an abnormal immune response which does not result in infiltration, or immune resistance, where the immune system is triggered but actively excluded from the site. While data do not exist for a definitive selection of the correct model, division of potential mechanisms into these three models provides a theoretical framework to design those experiments capable of distinguishing between them.

### Empirical Testing of *Candida* Biofilm Evasion Models Supports Immunological Resistance As the Dominant Mechanism

**Figure 2:**
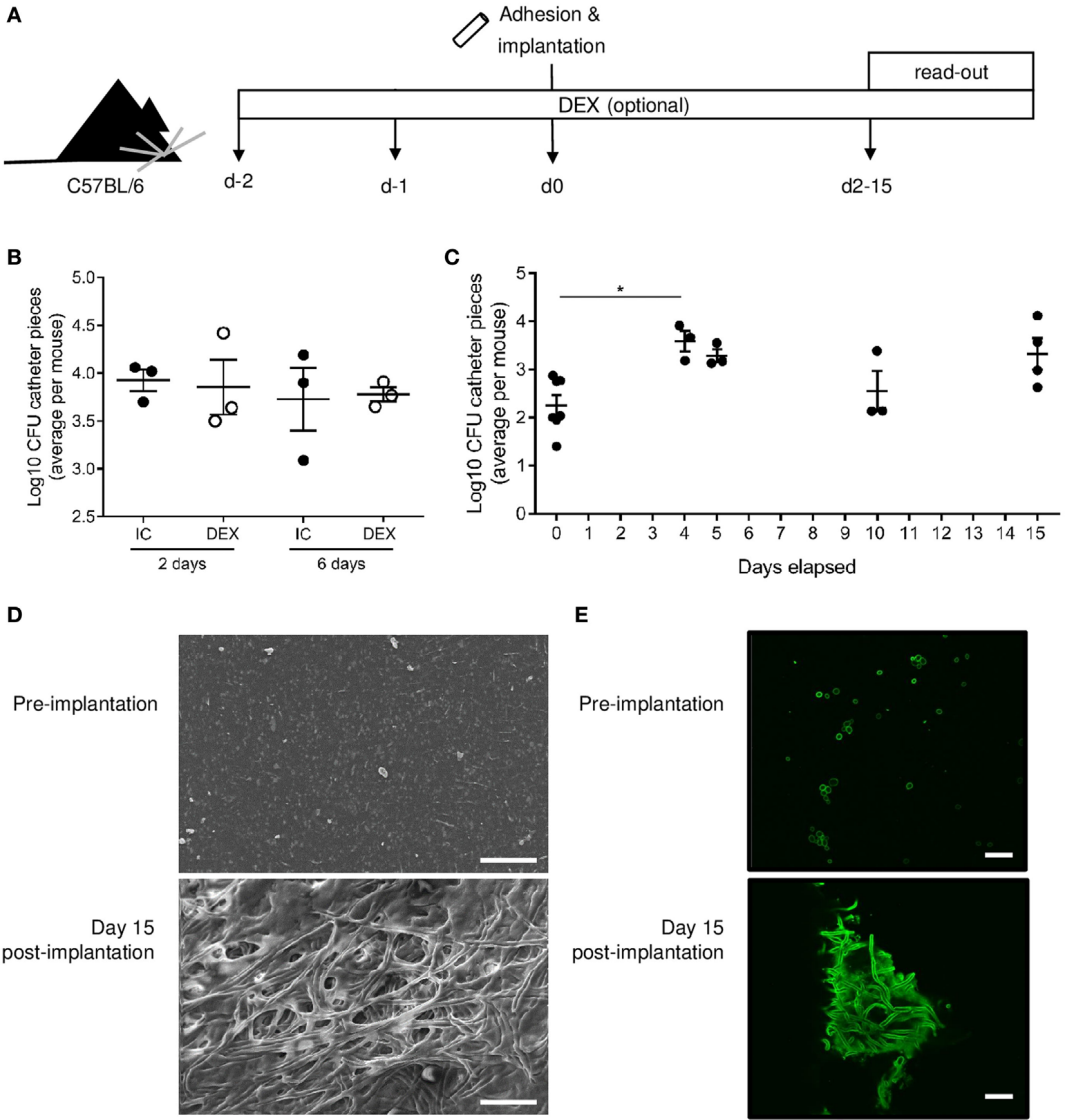
*Candida albicans* biofilms are robustly maintained in C57BL/6 mice without immunosuppression. **(A)** Catheter pieces were incubated overnight in mouse serum and for 90 min in *C. albicans* culture at day 0, following by subcutaneous implantation. In the dexamethasone-treated (DEX) group, dexamethasone (1 mg/L) was added in the drinking water from day 2 and maintained over the course of the experiment. Biofilm colony-forming units (CFUs) were read out at indicated days postimplantation. **(B)** Log_10_ of biofilm CFUs compared between immunocompetent (IC) and DEX mice (*n* = 3) at 2 and 6 days. Mean ± SEM are shown. **(C)** Log_10_ of biofilm CFUs followed over time (*n* = 3–6/group), day 0 = before implantation of catheter pieces. Mean and SEM are shown. One-way ANOVA with multiple comparisons was used for comparison of the different time points (*p < 0.05). **(D)** Representative scanning electron microscopy images of preimplantation (top), 2 days after implantation (middle), and day 15 after implantation (bottom). **(E)** Confocal laser scanning microscopy images of catheter pieces before implantation (top) and 15 days postimplantation (bottom). Scale bar = 20 µm.

Having developed a theoretical framework for the mechanisms by which *Candida* biofilms may evade clearance by the host immune system, we sought to formally distinguish between these possible modalities of host–pathogen relationships. Several mouse models have been developed to study *Candida* biofilms *in vivo*, typically utilizing immunosuppression (23, 24). As exogenous immunosuppression would prevent the utility of such a model for studying immune evasion, we developed a mouse model of *C. albicans* biofilm growth, which does not rely on immune suppression. *C. albicans* biofilms were grown on subcutaneous catheter pieces and implanted into both immunocompetent (IC) and dexamethasone-treated (DEX) mice, using the dose of dexamethasone required for previous immunosuppression-dependent models (Figure 2A). No major difference in the number of viable *Candida* recoverable from the biofilm was observed between the IC and DEX mice (Figure 2B), indicating a limited capacity of the immune system to clear biofilm infections within the 6-day window of observation. Furthermore, biofilm growth occurred in the first 4 days following implantation, with stable maintenance of viable *Candida* recoverable to at least 15 days postimplantation (Figure 2C). The maintained *C. albicans* biomass formed classical biofilms, visible by scanning electron microscopy (Figure 2D) and confocal laser scanning microscopy (Figure 2E). Despite the kidneys being the main infected organs in a systemic infection model, no colony-forming units (CFUs) were detected in kidneys of implanted mice (data not shown), indicating that *C. albicans*, when present in a biofilm on a subcutaneous catheter, does not release yeast form *Candida* capable of infecting distant organs. Together, the formation and robust maintenance of subcutaneous *C. albicans* biofilms in IC C57Bl/6 mice is consistent with the failure of the host immune system to eradicate biofilms in patients and provides a system for mechanistically testing the hypothesis of immune resistance of the biofilm without the limitations inherent to patient study.

We next developed an experimental setup capable of formally distinguishing the immune resistance model from the models of immune silence or deviation. It has been previously shown that a low intravenous (i.v.) exposure to yeast form *C. albicans* can protect mice from a subsequent lethal high-dose challenge (25). When these data are considered in our theoretical framework of immune evasion models, it provides an avenue for formal testing. The models of biofilm immune silence or immune deviation would postulate that *C. albicans* biofilms would either generate no immune response or an ineffective immune response, respectively, such that a biofilm exposure would not provide the protective effect generated by yeast-form exposure. The model of biofilm immunological resistance, by contrast, allows *C. albicans* biofilms to generate high-quality immune responses (which have no impact on the biofilm, due to intrinsic resistance mechanisms), which would nonetheless be capable of protection against a lethal yeast-form challenge.

**Figure 3:**
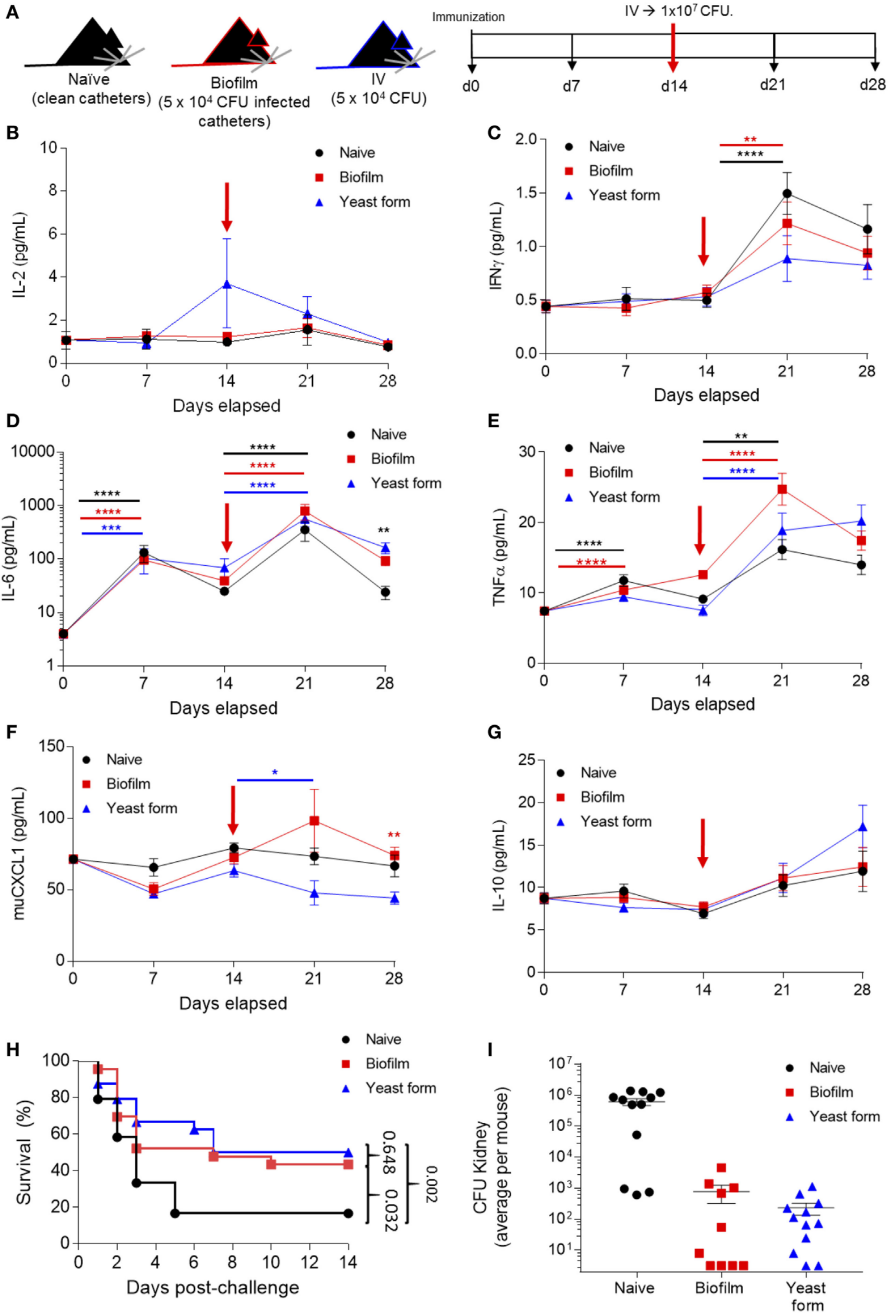
*Candida albicans* biofilm promotes sterilizing immunity against infection lethal dose of yeast form *C. albicans*. **(A)** Mice were surgically implanted with sterile catheters (“naïve” group, *n* = 24), with catheters seeded with 5 × 10^4^ *Candida* for biofilm growth (“biofilm immunized” group, *n* = 25) or i.v. injected with 5 × 10^4^ *Candida* (“yeast-form immunized” group, *n* = 25) on day 0. On day 14, each group was challenged with a 10^7^ colony forming unit (CFU) *Candida* i.v. Mice were monitored for cytokine expression on days 0, 7, 14, 21, and 28. **(B)** Serum samples from all the time points were analyzed for IL-2, **(C)** IFNγ, **(D)** IL-6, **(E)** TNFα, **(F)** muCXCL1, and **(G)** IL-10. Kruskal–Wallis test was used to compare the different groups at the same time point and to different time points. Average ± SEM. **(H)** Survival of all mice groups post high-dose challenge (1 × 10^7^ CFU). **(I)** Kidney colonization, showing the average count (Log_10_ CFU) per mouse on both kidneys after high-dose challenge for naïve mice (date of death, *n* = 12), biofilm immunized mice (*n* = 10, day 14 post-challenge), and yeast-form immunized mice (*n* = 12, day 14 post-challenge). Cytokine and survival data are pooled from two independent experiments (**p < 0.005, ***p < 0.0001, and ****p < 0.00001).

We therefore designed an immunization experiment to test the capacity of the *C. albicans* biofilm to induce protective immunity against the yeast form and thus distinguish immune resistance from immune silence or immune deviation. Mice were either left as naïve (implantation of clean catheters to control for surgery), implanted with a subcutaneous *C. albicans* biofilm formed from 5 × 10^4^ CFU per catheter, or i.v. immunized with 5 × 10^4^ yeast form *Candida* CFU. 14 days after low-dose infection, all mice were then challenged i.v. with 10^7^ CFU *C. albicans* (Figure 3A). Based on the three proposed models, it is expected that (i) if the biofilm is immunologically silent, mice inoculated with a low-dose *Candida* biofilm would behave like the naïve mice and present with a high-death rate; (ii) if the biofilm is presenting with immune deviation, mice inoculated with a low-dose *Candida* biofilm, would again behave like naïve mice, and have a similar or higher death rate, since the immune response being induced is deviated into an ineffective direction; and (iii) in the immune resistance model, mice inoculated with a low-dose *Candida* biofilm would behave like the mice inoculated with a low-dose i.v. *Candida*, have a low death rate since the biofilm is triggering trained immunity.

Using the biofilm/yeast-form immunization model and by taking serum samples at baseline, before surgery or i.v. infection, and at 7-day intervals, we were able to track the cytokine production as the infection progressed (Figures 3B–G). IL-6 and TNFα were significantly increased as a consequence of both the low-dose infection and the implantation of the catheters (measured at days 0 and 7) (Figures 3D,E). Following the high-dose challenge on day 14, 7 days later (day 21), we saw a significant increase in IFNγ, IL-6, and TNFα (Figures 3C–E) for all groups, indicating that the systemic inflammation regardless of prior exposure. The key immunological readout in this assay is protection from infection. Naïve mice were highly susceptible to the high-dose (10^7^) intravenous infection, with 60% of mice dying within 3 days, and ~90% dying by 6 days (Figure 3H). By contrast, as previously described, mice immunized with a 5 × 10^4^ i.v. *Candida* demonstrated protective immunity, with 50% survival out to day 14 (Figure 3H). Critically, mice receiving the same dose of *C. albicans* in a biofilm form showed the same protection against a lethal second dose in the yeast form (Figure 3H), formally demonstrating that *C. albicans* biofilms generate effective immune responses from the host, despite being resistant to the resulting immunity. At the level of yeast eradication, the immune response induced by both *C. albicans* biofilms and the low-dose yeast form was effective at reducing the infectious load, with sterilizing or near-sterilizing immunity in both groups at day 14 post-challenge, compared with the infectious load in end-stage naïve mice (Figure 3I). Together, these results formally demonstrate that biofilms promote efficient immunity in the host, capable of sterilizing immunity, and yet are resistant to immune clearance.

## Concluding Remarks

The ability of *Candida* biofilms to evade host immune clearance can be explained through three, not mutually exclusive, models. These models are (i) immunological silence, with the biofilm preventing immune sensing; (ii) immunological deviation, with the biofilm driving the immune response into a non-productive avenue; and (iii) immune resistance, with the biofilm no longer being sensitive to the same immunological attacks competent to clear non-biofilm forms. While research in the field often starts through identification of molecular pathways and then requires elucidation of the mechanism, clustering all potential pathways of immune evasion into these three basic models allows the reverse approach, where the general mechanism can first be understood, driving the identification of molecular pathway.

As proof-of-principle of the utility of this perspective, we designed experiments in which the different models proposed here would give unique outcomes. Using an animal model of *Candida* infection, we demonstrated that a subcutaneous biofilm can confer an immunological memory or trained immunity to the host, as evidenced by an increase in survival and the eradication of yeast from the kidney. This mouse experiment formally demonstrates that the biofilm is not immunologically silent, nor does it create a systemic immune deviation capable of inhibiting anti-*Candida* responses. Rather, the biofilm’s structure renders it unresponsive to immunological challenge, the “immune resistance” model. The biofilm structure functions to create an asymmetry in the relationship between host and microbe, with the immune system able to recognize and respond to the infection, but with the biofilm sheltering the pathogen from eradication. While our study does not attempt to identify the molecular determinants of this immune resistance, it provides a framework of the key characteristics on which future research can be based.

## Materials and Methods

### Mice

Animals used for the experiments were 8–10 weeks old female C57BL/6J mice bred in-house or purchased from Janvier Labs. Food and water were supplied *ad libitum*. Immunosuppression was carried out by dexamethasone in drinking water at 1 mg/L (26). Cytokine serum levels were quantified by an electrochemiluminescence immunoassay format using Meso Scale Discovery (Rockville, MD, USA) murine pro-inflammatory panel 1.

### Infection Models

*Candida albicans* strain SC5314 (27) was used for mouse infection studies. For growth and quantification details, see below. For immunization experiments, C57BL/6J were subcutaneously implanted with six clean catheter pieces or six *C. albicans* colonized catheter pieces, or injected intravenously with 5 × 10^4^ *Candida* CFU (see *Candida albicans* Growth and Quantification for culturing details). The same *Candida* preculture was used in the latter two and cultured as described for systemic infection. Day 14 after treatment, all mice received 10^7^ *C. albicans*. Takedown of surviving mice and catheter and kidney explant was performed at day 28.

### *C. albicans* Growth and Quantification

*Candida albicans* strain SC5314 (27) was maintained on YPD agar plates comprised 1% yeast extract (Merck), 2% bactopeptone (Oxoid), 2% glucose (Fluka analytics), and 1.5% Difco agar (BD). To quantify *C. albicans* biomass in kidneys, the kidneys were removed aseptically and homogenized (PRO250) in PBS. To quantify *C. albicans* biomass on catheter pieces, aseptically explanted catheter pieces were stored in 1× PBS, sonicated for 10 min at 40 kHz, and vortexed for 30 s. For each sample type, serial dilutions were made in PBS and plated in duplicate on YPD agar plates with 50 mg/L chloramphenicol. Plates were incubated at 37°C for 1–2 days. CFUs were calculated by taking the mean of both duplicates of the lowest dilution resulting in a countable number of colonies on the plate.

For subcutaneous biofilm infections, polyurethane triple-lumen intravenous catheters (Arrow International) were cut into pieces of 1 cm and incubated overnight in mouse serum (Sigma) at 37°C. *C. albicans* cell cultures were grown overnight in YPD liquid medium at 37°C, counted using a Bürker Chamber (Labor Optik), and the desired challenge inocula were prepared in RPMI-1640 medium (Sigma-Aldrich) buffered at pH 7.0 with 34.53 g/L MOPS (Sigma-Aldrich). Serum-coated catheter pieces were incubated for 90 min in 1 mL of cell suspension or in 1 mL of RPMI-MOPS. After incubation, catheter pieces were washed twice with PBS before being implanted. Surgery and explant of the catheter pieces was performed as described before (26, 28). Animals were anesthetized by injecting a cocktail of 45–60 mg/kg ketamine (Anesketin^®^) and 0.6–0.8 mg/kg medetomidine (Domitor^®^) in sterile saline i.p. Anesthesia was reversed by i.p. injection of 0.5 mg/kg atipamezole (Antisedan^®^) in sterile saline (28).

For systemic infection, a single *C. albicans* colony from a YPD agar plate was incubated at 30°C for 24 h was restreaked on a fresh YPD agar plate and grown at 30°C for another 24 h. A single colony from the latter was incubated in liquid YPD medium and grown for 12–14 h at 30°C shaking at 200 rpm. Cells were counted using the Bürker chamber; the desired inocula were prepared in a sterile saline solution and confirmed by plating. Mice were infected or mock infected (PBS) by injection in the lateral tail vein.

### Microscopy

Microscopy was modified from published protocols (26). Briefly, catheter pieces were cut longitudinally opening up the lumen and were allowed to air dry overnight. For scanning electron microscopy, mounted samples were sputter coated with Au–Pd and examined on an FEI XL30-FEG at 10 kV and 10 mm working distance. For confocal laser scanning microscopy, catheter pieces were cut longitudinally and stained using a 1/1,000 dilution of Alexa488-Concanavalin A in PBS. Samples were examined using an Olympus Fluoview FV1000 IX81.

### Statistics

All the groups were compared using Kruskal–Wallis test with a Dunn correction, or one-way ANOVA where stated. Survival curves were analyzed by Mantel–Cox test. Statistical analyses were performed using GraphPad Prism 6.04.

## Ethics Statement

All animal experiments were carried out by competent researches under the laboratory license LA1210570 and protocol number P122/2013, which was reviewed and approved by the KU Leuven animal ethical committee. Animal experiments adhered to the Belgian Royal Decree and the European Directive 2010/63/EU regulations regarding the protection and well-being of laboratory animals. All animal experiments were designed with the 3Rs guidelines.

## Author Contributions

Methodology and resources: JG-P, LM, SH-B, and AB. Validation, formal analysis, investigation, and writing-original draft: JG-P and LM. Writing review and editing: JG-P, LM, and AL. Supervision, project administration, and funding acquisition: AL, PD, and KL.

## Conflict of Interest Statement

The authors declare that the research was conducted in the absence of any commercial or financial relationships that could be construed as a potential conflict of interest.
